# 

**Published:** 1904-11

**Authors:** 


*Sixtieth Year.*

VOL. LX.
WHOLE NUMBER,
DCLXXXXVI.

NOVEMBER, 1904.

New Series.
VOL. XLIV
No. 4

INDEXED

### BUFFALO MEDICAL JOURNAL

Established 1845 by AUSTIN FLINT, M. D.

LIBRARY
[illegible] OFFICE
NOV. -3 1904

EDITOR:

WILLIAM WARREN POTTER, M. D.

ASSISTANT EDITORS:
WILLIAM C. KRAUSS, M. D. NELSON W. WILSON, M. D.

ASSOCIATE EDITORS:

JAMES WRIGHT PUTNAM, M. D. ERNEST WENDE, M. D.

JOHN PARMENTER, M. D. JOHN A. MILLER, PH. D.

HARVEY R. GAYLORD, M. D. MAUD J. FRYE, M. D.

